# Method for Indirect Quantification of CH_4_ Production via H_2_O Production Using Hydrogenotrophic Methanogens

**DOI:** 10.3389/fmicb.2016.00532

**Published:** 2016-04-29

**Authors:** Ruth-Sophie Taubner, Simon K.-M. R. Rittmann

**Affiliations:** ^1^Research Platform: ExoLife, University of ViennaVienna, Austria; ^2^Institute of Astrophysics, University of ViennaVienna, Austria; ^3^Archaea Biology and Ecogenomics Division, Department of Ecogenomics and Systems Biology, University of ViennaVienna, Austria

**Keywords:** Archaea, anaerobic cultivation, psychrophile, hyperthermophile, methanogenesis, water, methane, standard operation procedure (SOP)

## Abstract

Hydrogenotrophic methanogens are an intriguing group of microorganisms from the domain Archaea. Methanogens exhibit extraordinary ecological, biochemical, and physiological characteristics and possess a huge biotechnological potential. Yet, the only possibility to assess the methane (CH_4_) production potential of hydrogenotrophic methanogens is to apply gas chromatographic quantification of CH_4_. In order to be able to effectively screen pure cultures of hydrogenotrophic methanogens regarding their CH_4_ production potential we developed a novel method for indirect quantification of the volumetric CH_4_ production rate by measuring the volumetric water production rate. This method was established in serum bottles for cultivation of methanogens in closed batch cultivation mode. Water production was estimated by determining the difference in mass increase in a quasi-isobaric setting. This novel CH_4_ quantification method is an accurate and precise analytical technique, which can be used to rapidly screen pure cultures of methanogens regarding their volumetric CH_4_ evolution rate. It is a cost effective alternative determining CH_4_ production of methanogens over CH_4_ quantification by using gas chromatography, especially if applied as a high throughput quantification method. Eventually, the method can be universally applied for quantification of CH_4_ production from psychrophilic, thermophilic and hyperthermophilic hydrogenotrophic methanogens.

## 1. Introduction

Methanogens are an intriguing group of microorganisms with extraordinary ecological (Liu and Whitman, 2008), biochemical (Ferry, 2010; Thauer et al., 2010), physiological (Thauer et al., 2008; Taubner et al., 2015) characteristics, and promising biotechnological potential (Seifert et al., 2013, 2014; Rittmann et al., 2015; Rittmann, 2015). Currently all methanogens are phylogenetically classified into the phylum Euryarchaeota (Liu and Whitman, 2008; Borrel et al., 2013) but recently the metabolism of a putative methane (CH_4_) producing *Candidatus* lineage was reconstructed *in silico* and assigned to the phylum Bathyarchaeota (Evans et al., 2015).

Methanogens can be found in almost every anoxic environment, e.g., lakes (Franzmann et al., 1997), sediments (Schirmack et al., 2014), soils (Lü and Lu, 2012; Wagner et al., 2013), bog, fen and palsa of thawing permafrost (Mondav et al., 2014), sea floor (Jones et al., 1983; Jeanthon et al., 1999; von Klein et al., 2002; L'Haridon et al., 2003), and anaerobic digesters (Jiang et al., 2005; Ma et al., 2006; Parshina et al., 2014; Rittmann and Holubar, 2014). Methanogens are the only anaerobic organisms known to date that are capable of producing CH_4_ as an end product of their energy conserving metabolism. Therefore, they play an important role in the terrestrial global carbon cycle (Liu and Whitman, 2008; Thauer et al., 2008; Mondav et al., 2014).

Methanogens are obligate chemolithoautotrophic or chemolithoheterotrophic organisms, which obtain cellular carbon and energy from C_1_-, C_2_-, and methylated compounds—either by reduction with molecular hydrogen (H_2_) or by using a disproportionation reaction. Furthermore, some methanogens are known to metabolize carbon monoxide (Oelgeschläger and Rother, 2009; Ferry, 2010). A physiological characteristic of methanogens is that they may vary their growth to product yield (Y_(*x*/*CH*4)_) upon experiencing a change in environmental conditions—a mechanism commonly referred to as uncoupling (Mountfort and Asher, 1979; Archer, 1985; Fardeau and Belaich, 1986; Tsao et al., 1994; Liu et al., 1999; Pennings et al., 2000; Ver Eecke et al., 2013; Bernacchi et al., 2014; Taubner et al., 2015).

In biotechnology, methanogens are integral members of the biocoenosis applied for biogas production in anaerobic digesters and they are commonly used in anaerobic waste water treatment plants. Furthermore, methanogens are utilized for biological CH_4_ production in pure culture—a process also referred to as biomethanization. Especially hydrogenotrophic, autotrophic and thermophilic methanogens were found to exhibit extraordinary strain specific (e.g., specific growth rate (μ) and high specific CH_4_ evolution rates Rittmann et al., 2012) and industrially relevant characteristics, such as high volumetric CH_4_ production rates (MER) (Seifert et al., 2014).

Hydrogenotrophic methanogens utilize H_2_ as the electron donor for the reduction of carbon dioxide (CO_2_) to CH_4_. These organisms produce CH_4_ and water (H_2_O) as metabolic products according to the following stoichiometric reaction (Liu and Whitman, 2008; Thauer et al., 2008; Rittmann et al., 2015):

(1) $$4\;H_{2}+CO_{2}\to CH_{4}+2\;H_{2}O\;\Delta {\text{G}}^{{}^{\circ}}=-135\text{ kJ}/{\text{mol CH}}_{4}.$$

The standard method to determine the CH_4_ production potential of methanogens is to perform gas chromatographic measurements. The aim of this study was, however, to establish a cheaper, faster and at the same time a precise and accurate method to determine the CH_4_ production potential of hydrogenotrophic methanogens grown in serum bottles via H_2_O production.

The presented method is based on the following rationale: due to the conversion of four moles H_2_ and one mole CO_2_, one mole of CH_4_ and two moles H_2_O are produced, which leads to a decrease of serum bottle headspace pressure and a concomitant increase of liquid body mass due to H_2_O formation. Based on this principle, we present a novel method for quantification of both the CH_4_ production and the mass gain by determining the pressure reduction inside the serum bottle and the water accumulation, respectively—in one simple procedure.

## 2. Materials and methods

We examined four different strains of hydrogenotrophic and autotrophic methanogens (*Methanosarcina soligelidi* DSM 26065, *Methanothermococcus okinawensis* DSM 14208, *Methanocaldococcus villosus* DSM 22612, and *Methanothermobacter marburgensis* DSM 2133) (see Table 1). The strains were grown in 120 mL serum bottles (La-Pha-Pack, Langerwehe, Germany) in chemically defined media (see Section 2.1). The inoculation was performed inside an anaerobic chamber (Coy Laboratory Products, Grass Lake, USA). Residual CH_4_ was replaced in regular intervals with a CO_2_/H_2_ test gas mixture (20 Vol.-% CO_2_ in H_2_) pressurizing each of the serum bottles to 1.4–2.0 barg.

**Table 1:** **Overview of methanogens and cultivation settings used in this study**.

**Strain DSMZ number**	***M. marburgensis* DSM 2133**	***M. villosus* DSM 22612**	***M. okinawensis* DSM 14208**	***M. soligelidi* DSM 26065**
Volume (medium) [mL]	50	50	50	50
	45			
Incubation temperature [°C]	55	80	65	28
	65			
Gas pressure [barg]	1.4–2.0	1.5–2.0	1.4–1.7	1.4–1.6
Gassing interval	daily	1–2 times a day	daily	Every fifth day
Number of incubation periods per experiment	8	8–9	8	7

### 2.1. Media

The medium for the cultivation of *M. marburgensis* contained 2.1 g NH_4_Cl, 6.8 g KH_2_PO_4_, 3.6 g Na_2_CO_3_, 5 mL of 200 × trace element solution, and was filled up to 1 L with ddH_2_O. For the 200 × trace element solution first 9.0 g Titriplex I was dissolved in 400 mL ddH_2_O. A pH of 6.5 was adjusted by adding 5M NaOH. Then 4.0 g of MgCl_2_ · 6H_2_O, 1.0 g FeCl_2_ · 4H_2_O, 20 mg CoCl_2_ · 6H_2_O, 120 mg NiCl_2_ · 6H_2_O, and 20 mg NaMoO_4_ · 2H_2_O were added. A pH of 7.0 was adjusted by adding 1M NaOH and the volume of the trace element solution was filled up with ddH_2_O to 500 mL. 50 mL of the medium were aliquoted into 120 mL serum bottles and sealed with blue rubber stoppers (pretreated by boiling ten times for 30 min in fresh ddH_2_O; 20 mm, butyl rubber, CLS-3409-14, Chemglass Life Sciences) and crimp caps (20 mm aluminum, Ochs Laborbedarf, Bovenden, Germany). After anaerobization by five times gassing (0.8 barg) and four times drawing vacuum, the serum bottles were autoclaved. The last step of preparation was to anaerobically add 0.1 mL of sterile 0.5 M Na_2_S · 9 H_2_O to the serum bottles in an anaerobic chamber.

For *M. villosus* and *M. okinawensis*, the DSMZ Medium 282 (2014) was used, with the following modifications: 0.14 g K_2_HPO_4_ was substituted with 0.183 g K_2_HPO_4_ · 3 H_2_O and 0.01 g Fe(NH_4_)_2_(SO_4_)_2_ · 6 H_2_O was replaced with 7 mg FeSO_4_ · 7 H_2_O and we omitted the resazurin solution from the medium. The sterile filtered vitamin solution (modified from Balch et al., 1979) contained per L 20 mg biotin, 20 mg folic acid, 100 mg pyridoxamine dihydrochloride, 50 mg thiamine hydrochloride, 50 mg riboflavin, 50 mg niacin, 50 mg calcium-D(+)-pantothenat, 5 mg cyanocobalamin, 50 mg para-aminobenzoic acid, and 25 mg lipoic acid. Medium aliquots were prepared by adding 50 mL into 120 mL serum bottles. The bottles were sealed with blue rubber stoppers and crimp caps. After anaerobization by five times gassing (0.8 barg) and four times drawing vacuum, the serum bottles were autoclaved. Thereafter, 0.5 mL of the two times sterile filtered aforementioned vitamin solution was added aseptically to the serum bottles. Eventually, 0.75 mL of sterile NaHCO_3_ solution (of 3 g NaHCO_3_ in 45 mL ddH_2_O), 0.25 mL of sterile L-Cysteine-HCl · H_2_O (solution 10 g L-Cysteine-HCl in 100 mL ddH_2_O) and 0.2 mL of sterile 0.5 M Na_2_S · 9 H_2_O were added to the serum bottles in an anaerobic chamber.

For *M. soligelidi*, we used the medium described by Wagner et al. (2013) with the following modifications: 0.8 g KH_2_PO_4_ · 2 H_2_O was substituted with 0.633 g KH_2_PO_4_ and we omitted the resazurin solution from the medium. For this medium we used the aforementioned vitamin solution and the trace element solution as reported by Balch et al. (1979). For the trace element solution we substituted 0.5 g MnSO_4_ · 2 H_2_O with 0.529 g MnCl_2_ · 4 H_2_O, 0.1 g CoCl_2_ with 0.183 g CoCl_2_ · 6 H_2_O, 0.1 g ZnSO_4_ with 0.178 g ZnSO_4_ · 7 H_2_O, 0.01 g CuSO_4_ · 5 H_2_O with 0.006 g CuSO_4_, and 0.01 g AlK(SO_4_)_2_ with 0.018 g AlK(SO_4_)_2_ · 12 H_2_O. After anaerobization by five times gassing (0.8 barg) and four times drawing vacuum, the serum bottles were autoclaved. The next step was to add 0.5 mL of the two times sterile filtered vitamin solution into each of the serum bottles. Finally, 0.2 mL of sterile 0.5 M Na_2_S · 9 H_2_O were anaerobically added to the serum bottles in an anaerobic chamber.

### 2.2. Culture setup

Before starting the experiments, the mass of the empty 120 mL serum bottles, the crimp caps, and the blue rubber stoppers has to be determined to quantify the exact amount of medium added afterwards. The balance should be capable to accurately weigh a difference in mass in the range of 0.1 mg (used balance here: AT261 Delta Range Analytical Balance, Mettler Toledo, USA). Based on these data we could calculate the respective gas volume in the serum bottles. All experiments were performed at least in triplicates plus two negative controls. The first negative control is treated like an inoculated flask (cultivation control) and the second is needed to determine the pressure in the serum bottles after the flush and purge process (gas control). For gassing, a gassing manifold was used (see **Figures 3B,C**). We used 3-way stopcocks (PSU, RED, 8501742, Fresenius Kabi AG, Germany), filters (sterile syringe filters, w/0.2c μm cellulose, 514-0061, VWR International, USA)), and cannulae (disposal hypodermic needle, Gr 14, 0.60 × 30 mm, 23 G × 1 1/4″, RX129.1, Braun, Germany) to add the H_2_/CO_2_ test gas to the serum bottles. To purge the gas phase, we opened the 3-way stopcocks one by one for 2–4 s so that the gas inside the bottle could be released. To measure the serum bottle headspace pressure we used digital manometer (LEO1-Ei, −1…3bar rel, Keller, Germany).

#### 2.2.1. Inoculation

We inoculated the serum bottles with 1 mL (2 Vol.-%) of a respective pre-culture in exponential growth phase. The negative controls were inoculated with 1 mL of the respective fresh medium. The inoculation was performed in an anaerobic chamber. After inoculation the serum bottles were weighed again to determine the exact amount of inoculum. Subsequently, the serum bottles were pressurized with H_2_/CO_2_ (1.4–2.0 barg depending on the strain) as gaseous substrates and weighted again. Cultures were incubated either in a waterbath (*M. marburgensis* and *M. okinawensis*) or in an air bath (*M. soligelidi* and *M. villosus*) in the dark at their respective optimal temperatures (see Table 1, the temperature could vary ± 2°C).

### 2.3. Standard operation procedure (SOP)

The SOP outlined here describes the workflow for quantification of CH_4_ production by using H_2_O production. This routine has to be applied for each sampling and gassing round (further referred to as an experimental run). Each experiment consists of seven to ten experimental runs. The incubation period between these runs should be long enough so that sufficient methanogenesis can take place (e.g., for *M. marburgensis* about 15–20 h).

**Figure 1:**
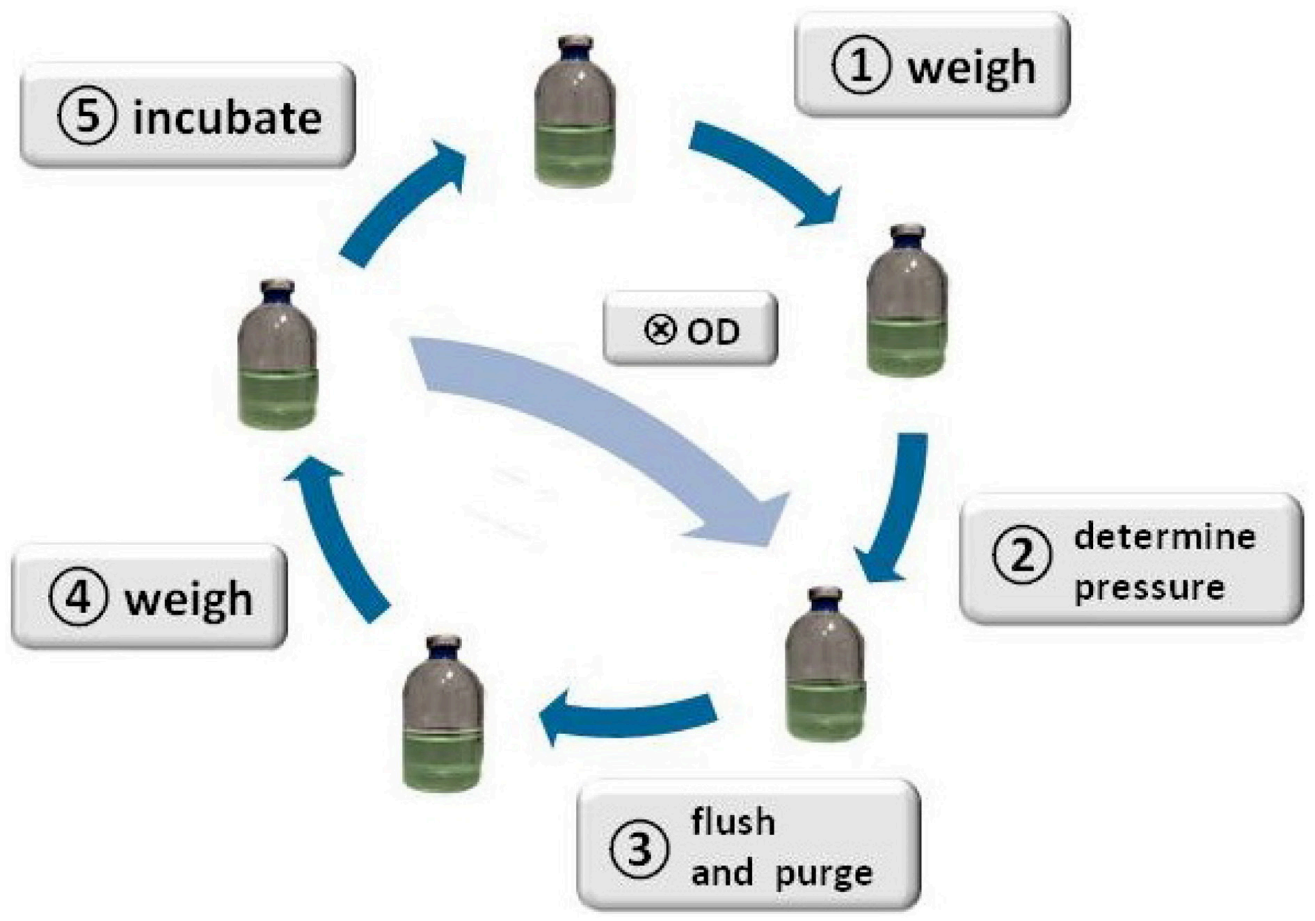
**Schematic illustration of the method**. The figure illustrates the SOP: start with weighing the serum bottles ①, determine the head space pressure of the serum bottles②, flush and purge to remove CH_4_ from the headspace of the serum bottles③, weigh the serum bottles ④, incubate the serum bottles ⑤, start again with step ①; taking a sample Ⓧ for OD measurement or cell counting is optional after step ④, whereas step ③ and ④ would have to be repeated after sampling for OD measurement.

1. Preparation
  - take the flasks out of the waterbath/airbath and dry the bottles with a tissue. Let them cool down to room temperature for approximately 1 h separated from one another by at least 15 cm distance (note the time)
2. Weight measurement
  - note room temperature and time
  - check if the balance is properly adjusted (check spirit level)
  - turn on the balance
  - tare the balance
  - measure the weight of each flask and note the values (Figure 1, step ①)
  - note room temperature and time
3. Pressure measurement and gassing
  - note room temperature and time
  - ensure that all globe valves are closed
  - clean the bench and the globe valves with *EtOH* (70 Vol.-%)
  - if not already in place, put a 3-way stopcock, a filter, and a cannula as can be seen in **Figure 3B** on to the H_2_/CO_2_-ports; ensure, that the 3-way stopcocks are half open: 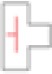  - measure the pressure of each flask with a digital manometer and note the values and time (Figure 1, step ②). For that purpose, put every time some *EtOH* (70 Vol.-%) on the rubber, wait until the *EtOH* (70 Vol.-%) is evaporated, and introduce the manometer-cannula and filter.
  - Remove the manometer-cannula and filter. Note if a droplet escapes while removing the manometer.
  - open the primary globe valve and adjust the pressure on the secondary valve (~1.4–2.0 bar)
  - Gassing the flasks:
    1. put *EtOH* (70 Vol.-%) on all serum bottles and the reference flask
    2. open the distribution valve
    3. open the first H_2_/CO_2_-port valve and introduce the prepared H_2_/CO_2_-port cannula into the negative control flasks—repeat this step for the remaining flasks
    4. wait ~1 min
    5. flushing the headspace (see Figure 1, step ③): switch the position of the valve on the 3-way stopcock for 2–4 s: 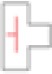 → 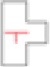 → 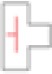, repeat this step for the remaining serum bottles
    6. repeat the previous step (5) another two times
    7. wait ~3 min - in the meantime swirl the flasks slightly several times
    8. close the distribution valve
    9. close the H_2_/CO_2_-port valves as simultaneously as possible
    10. pull out the H_2_/CO_2_-port cannula carefully and note if a droplet escaped while removing the cannula
    11. put new cannulae on the H_2_/CO_2_-ports or close the cannulae carefully
    12. measure the pressure in the reference flask as described in 3e and note the value
  - close the primary globe valve, open one of the unused H_2_/CO_2_-ports to release the pressure, close the secondary valve, close the H_2_/CO_2_-port valve
  - note room temperature and time
4. Weight measurement
  - repeat steps (2a to 2f) (Figure 1, step ④)
5. Finish
  - put the flasks in the corresponding waterbath or airbath for incubation (Figure 1, step ⑤)
  - note the time

**Figure 2:**
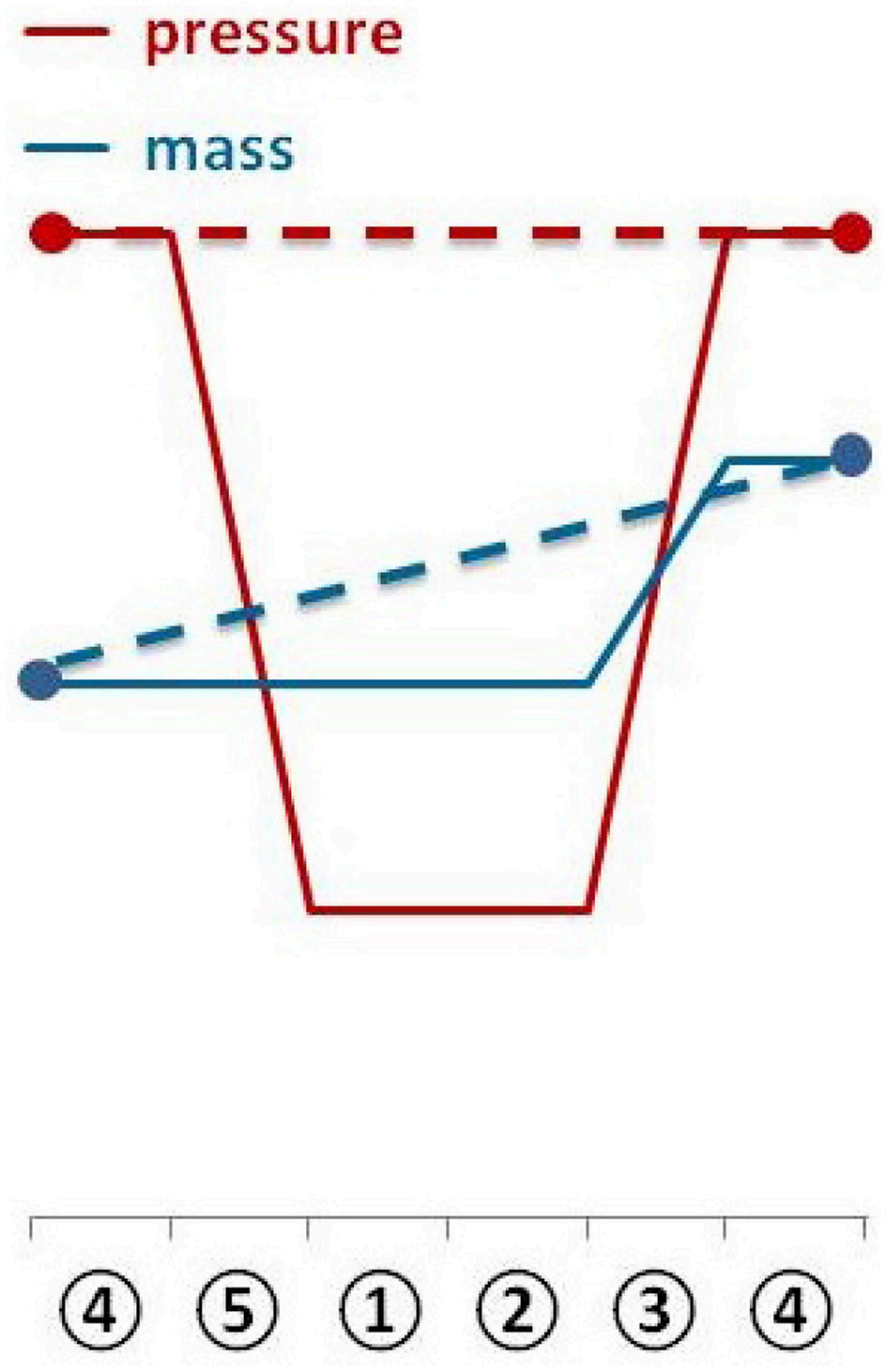
**The diagram shows the change of serum bottle mass and serum bottle headspace pressure during one experimental run**.

**Figure 3:**
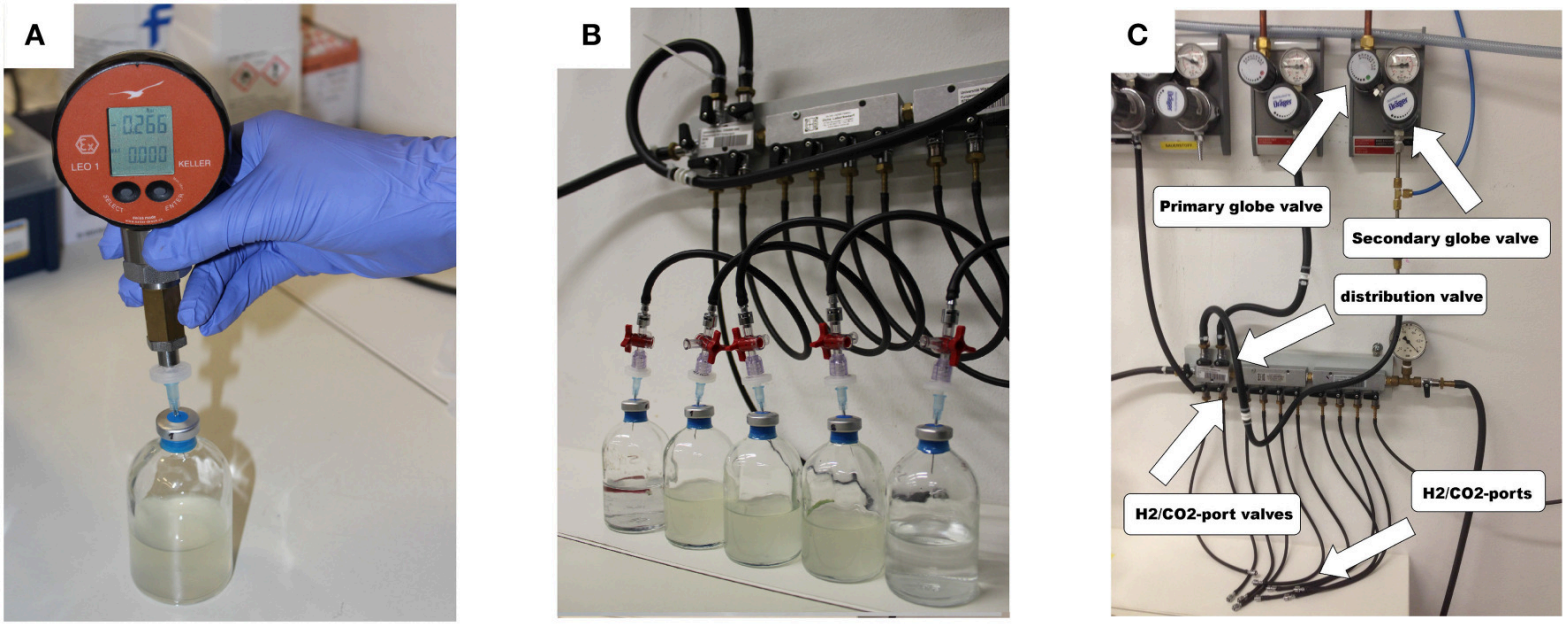
**(A)** Measurement of the serum bottle headspace pressure with a digital manometer; **(B)** parallel gassing of the serum bottle headspace; **(C)** gassing manifold.

Applying almost isothermal conditions during the determination of the headspace gas pressure in every step of the method is important in order to assure that the change in the headspace gas pressure is not caused by the varying temperature of the serum bottles or the medium, but due to the conversion of H_2_/CO_2_ to CH_4_. Before gassing, the weight of the serum bottles should be the same as at the end of the previous run because in a closed system the mass stays constant. However, the pressure in the inoculated flasks should have dropped during incubation due to the ongoing process of methanogenesis (see Figure 2). To receive the same gas composition as on the day before, we flushed and purged the serum bottles with H_2_/CO_2_ (see Figure 1, step ③). This step is important in order to achieve a nearly isobaric setting in which it is possible to determine the increase in weight due to the production of H_2_O. In other words, we removed the produced CH_4_ and residual H_2_/CO_2_ and added new H_2_/CO_2_. The flush and purge routine was done in a parallel setting for all serum bottles of one experimental run at once (see Figure 3B). It is important to do this step carefully and attentively to avoid surrounding air entering or medium exiting the serum bottle. After the last purging (step 3.h(7)), the flasks were flushed for at least 3 min to establish roughly the same pressure in all of the flasks. After gassing, we measured the actual pressure of the gas control serum bottle to assess the amount of pressure in the serum bottles. Later, this value will be used to calculate the respective conversion factor. The last step was to measure the mass of the serum bottles again. At this point, the increase in mass of the inoculated serum bottles should be observable (10–60 mg, depending on the strain). The routine was repeated seven to nine times (see Table 1). The whole experimental procedure is illustrated in Figures 1 and 2. The duration of each individual step of the SOP should be kept constant in each consecutive run to acquire reproducible results.

During some experiments, we additionally took samples to measure the optical density (OD, λ = 578 nm, blanked with ddH_2_O) at each run. The sampling took place between step ④ and ⑤, whereas steps ③ and ④ were repeated after sampling.

### 2.4. Gas chromatography

Analysis of the headspace gas composition (CO_2_, H_2_, and CH_4_) of selected serum bottles was determined at the end of the final incubation period by using a gas chromatograph (7890A GC System, Agilent Technologies, Santa Clara, USA) equipped with a TCD detector and a 19808 ShinCarbon ST Micropacked Column (Restek GmbH, Bad Homburg, Germany). Autosampling of serum bottle headspace and concomitant gas injection into the gas chromatograph was accomplished by using a gas injection and control unit (Joint Analytical Systems GmbH, Moers, Germany).

## 3. Theory

To estimate the produced particle number of CH_4_ in the gas phase after each incubation, the partial pressure of CH_4_ has to be calculated. For that, Equation (1) has to be considered. Due to the stoichiometry presented in Equation (1), the partial pressure of CH_4_ is equal to one fourth of the difference in pressure before and after incubation (see Equation (2)). Eventually, with the aid of the ideal gas law, the number of CH_4_ in mol can be determined. This directly leads to the theoretical amount of produced H_2_O, because, according to Equation (1), the production of one mole CH_4_ is accompanied by the production of two moles of H_2_O.

We included the varying gas pressure during the flushing process as well as the potential loss of liquid due to the leakage of the rubber stoppers into our calculations. For further information please refer to the Supplementary Material.

The MER, volumetric H_2_ uptake rate (HUR), and volumetric CO_2_ uptake rate (CUR) are the differences in the number of millimoles of CH_4_, H_2_, and CO_2_, respectively, per time passed in hours and per medium volume in liters. The WER is the gain in mass in g per incubation time passed in hours and per volume in liters and divided by the molar mass of H_2_O. The following equations show how the different variables were calculated. For those experiments where the OD was not measured, the contribution of the biomass (i.e., Δ*x*, *r*_*x*_, and *Y*_(*x*/*CO*2)_) had to be neglected.

(2) $$p_{CH4}=\frac{p_{\text{before}}-p_{\text{after}}}{4}\;[\text{Pa}]$$

(3) $$\text{MER}=\frac{\Delta n_{CH4}}{\Delta t\;\cdot \;V}\;[{\text{mmol h}}^{-1}{\text{ L}}^{-1}]$$

(4) $$\text{HUR}=\frac{\Delta n_{H2}}{\Delta t\;\cdot \;V}\;[{\text{mmol h}}^{-1}{\text{ L}}^{-1}]\;$$

(5) $$\text{CUR}=\frac{\Delta n_{CO2}}{\Delta t\;\cdot \;V}\;[{\text{mmol h}}^{-1}{\text{ L}}^{-1}]$$

(6) $$\text{WER}=\frac{\Delta m_{H2O}}{\Delta t\;\cdot \;18.01528\;\cdot \;V}\;[{\text{mmol h}}^{-1}{\text{ L}}^{-1}]$$

(7) $$x=OD\;\cdot \;0.31\;[{\text{gL}}^{-1}]$$

(8) $$r_{x}=\frac{\Delta x}{\Delta t\;\cdot \;30.97}\;[{\text{C-mmol h}}^{-1}{\text{ L}}^{-1}]$$

(9) $$Y_{\left(CH4/CO2\right)}=\frac{\text{MER}}{\text{CUR}}$$

(10) $$Y_{\left(x/CO2\right)}=\frac{r_{x}}{\text{CUR}}$$

(11) $$\text{C-balances = }Y_{\text{(}CH\text{4/}CO\text{2)}}\text{ + }Y_{\text{(}x/CO\text{2)}}$$

(12) $$\text{H-balances}=\frac{1.879\;\cdot \;r_{x}+2\;\cdot \text{ WER}+4\;\cdot \text{ MER}}{2\;\cdot \text{ HUR}}$$

(13) $$\text{DoR}=\frac{4.24\;\cdot \;r_{x}+8\;\cdot \text{ MER}}{2\;\cdot \text{ HUR}}$$

The values used in the Equations (8) and (13) were taken from Bernacchi et al. (2014). The values in Equations (7) and (12) were experimentally determined. These values were determined for *M. marburgensis* and therefore could vary for other methanogens (see Section 5.1 and 5.3).

## 4. Results

### 4.1. Mass gain

**Figure 4:**
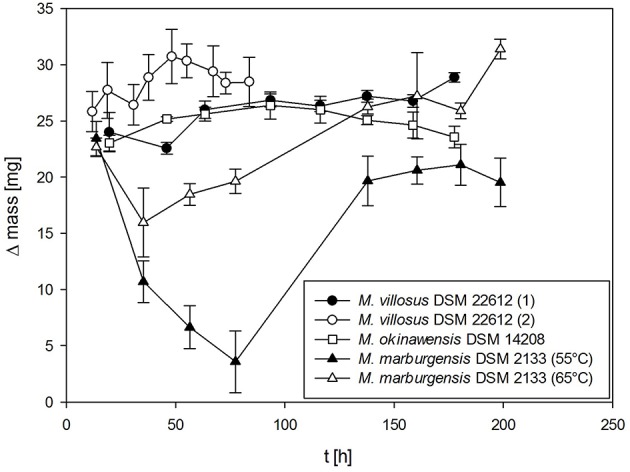
**Mass gain for ***M. villosus, M. okinawensis*** and ***M. marburgensis*****. For *M. villosus* an experiment with a daily gassing event [marked *M. villosus* (1)] and one experiment with two gassing events per day [marked as *M. villosus* (2)] were performed. For *M. marburgensis* cultivations at 55°C and at 65°C were performed. Negative controls are not shown.

**Figure 5:**
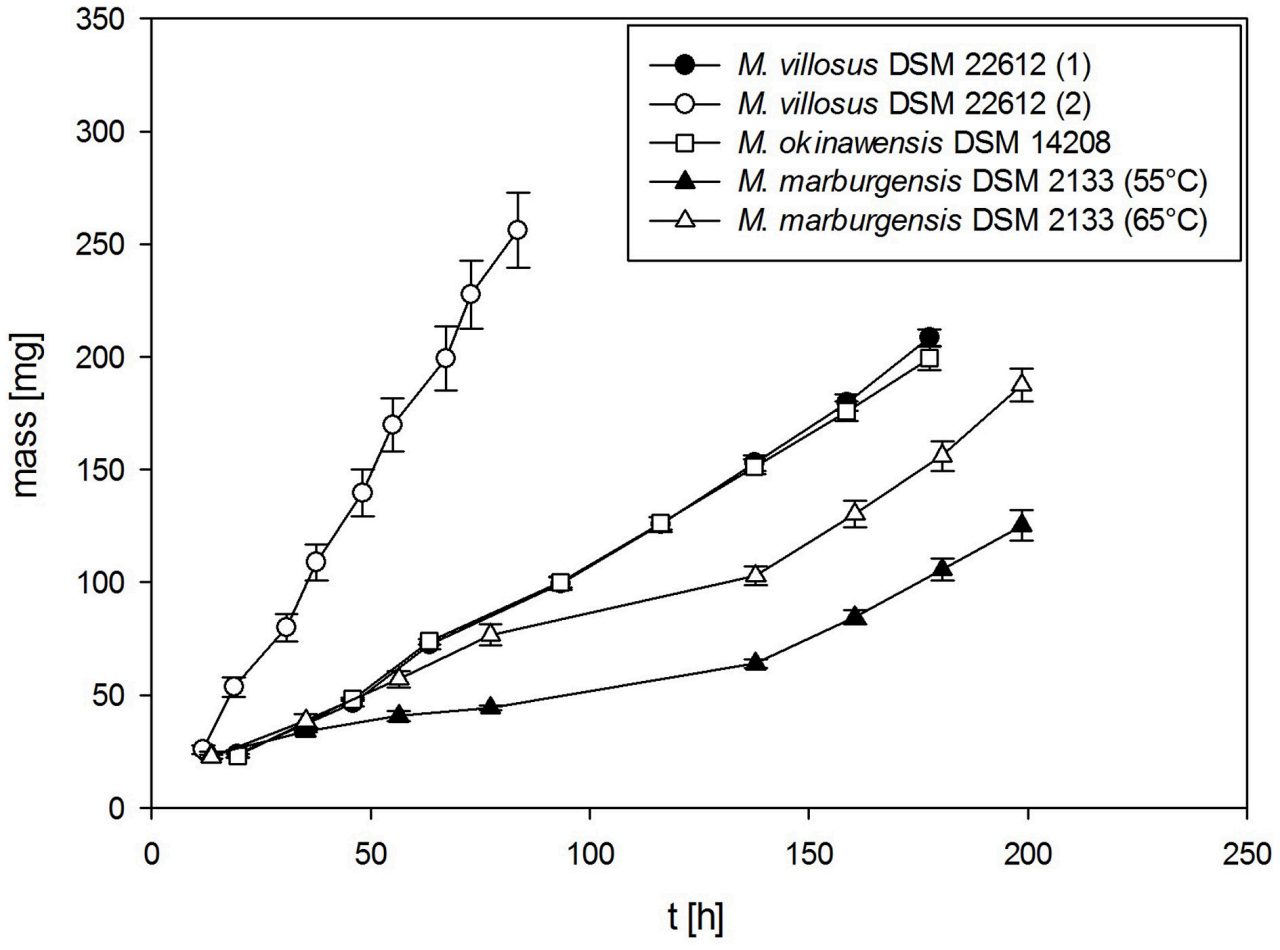
**Total cumulative mass gain for ***M. villosus***, ***M. okinawensis*** and ***M. marburgensis*****. For *M. villosus* an experiment with a daily gassing event [marked *M. villosus* (1)] and one experiment with two gassing events per day [marked as *M. villosus* (2)] were performed. For *M. marburgensis* cultivations at 55°C and at 65°C were performed. Negative controls are not shown.

The absolute mass gain of the different strains lies in average between 10 mg for *M. soligelidi* (please refer to Supplementary Material) and more than 25 mg for *M. villosus* (see Figure 4) per run. For about ten runs this sums up to a total mass gain of more than 80–250 mg of H_2_O per serum bottle (see Figure 5). The cumulative mass gain is dependent on the gassing frequency and the mass of convertible substrates in the headspace of the serum bottle. The low mass gain of *M. soligelidi* can be attributed to the short incubation time of 5 days (Supplementary Material). Additional experiments with a longer incubation period would need to be performed. The results for *M. villosus* and *M. soligelidi* show that this method can be used to easily determine the minimum incubation period necessary until a strain has performed full H_2_/CO_2_ conversion to CH_4_ by analysing the headspace gas pressure in parallel to the quantification of the H_2_O production.

Additionally, we varied the temperature (45, 55, and 65°C) when analysing the WER for *M. marburgensis*. At 45°C no growth could be observed for *M. marburgensis*. Furthermore, we varied the incubation time for *M. villosus* [routine performed daily (marked with *M. villosus* (1)] and two times a day [marked with *M. villosus* (2), respectively].

### 4.2. Mass balances

In Tables 2, 3 an overview of the results of the experiments is presented. The different values for *Y*_(*H*2*O*/*CH*4)_ and the mass balances are listed for every specific run of each experiment at the respective time. The mass balances were calculated according to the equations shown in Section 3. In Table 2 the mass balances for the experiments without OD sampling are shown, while in Table 3 the balances for the experiments with OD sampling are presented. For the calculated mass balances presented in columns 3–5 of Table 3 the biomass production rate was neglected, while for the mass balances given in column 6–8 the biomass production rate was taken into account. For detailed information about the calculation, please refer to the source code in the Supplementary Material.

**Table 2:** **Mass balances of the different experiments without OD measurements**.

**Strain**	**t [h]**	***Y*_(*H*2*O*/*CH*4)_**	**C-balance (%)**	**H-balance (%)**	**DoR-balance (%)**
*M. marburgensis DSM 2133 (55°C)*	13.68	11.95	92.39	92.38	92.38
*M. marburgensis DSM 2133 (55°C)*	35.23	12.33	187.45	187.25	187.25
*M. marburgensis DSM 2133 (55°C)*	56.45	3.00	119.90	119.93	119.93
*M. marburgensis DSM 2133 (55°C)*	77.35	0.62	96.20	96.20	96.20
*M. marburgensis DSM 2133 (55°C)*	137.90	2.76	95.27	95.25	95.25
*M. marburgensis DSM 2133 (55°C)*	160.57	1.25	98.35	98.35	98.35
*M. marburgensis DSM 2133 (55°C)*	180.47	2.29	97.40	97.41	97.41
*M. marburgensis DSM 2133 (55°C)*	198.55	1.86	99.86	99.86	99.86
*M. marburgensis DSM 2133 (65°C)*	13.68	11.66	94.10	94.09	94.09
*M. marburgensis DSM 2133 (65°C)*	35.23	1.83	105.08	105.08	105.08
*M. marburgensis DSM 2133 (65°C)*	56.45	1.11	102.26	102.26	102.26
*M. marburgensis DSM 2133 (65°C)*	77.35	1.22	99.52	99.52	99.52
*M. marburgensis DSM 2133 (65°C)*	137.90	1.70	98.98	98.98	98.98
*M. marburgensis DSM 2133 (65°C)*	160.57	1.63	99.52	99.52	99.52
*M. marburgensis DSM 2133 (65°C)*	180.47	1.59	99.27	99.26	99.26
*M. marburgensis DSM 2133 (65°C)*	198.55	1.84	99.94	99.94	99.94
*M. villosus DSM 22612 (1)*	19.68	1.39	97.07	97.06	97.06
*M. villosus DSM 22612 (1)*	45.93	1.28	100.38	100.37	100.37
*M. villosus DSM 22612 (1)*	63.45	1.50	98.06	98.05	98.05
*M. villosus DSM 22612 (1)*	93.35	1.51	100.23	100.22	100.22
*M. villosus DSM 22612 (1)*	116.15	1.43	102.28	102.27	102.27
*M. villosus DSM 22612 (1)*	137.67	1.52	100.02	100.02	100.02
*M. villosus DSM 22612 (1)*	158.65	1.50	100.28	100.28	100.28
*M. villosus DSM 22612 (1)*	177.53	1.61	100.44	100.44	100.44
*M. villosus DSM 22612 (2)*	11.83	1.50	93.02	93.03	93.03
*M. villosus DSM 22612 (2)*	18.82	1.52	99.65	99.65	99.65
*M. villosus DSM 22612 (2)*	30.82	1.41	102.05	102.05	102.05
*M. villosus DSM 22612 (2)*	37.62	1.56	100.40	100.39	100.39
*M. villosus DSM 22612 (2)*	48.20	1.65	100.63	100.63	100.63
*M. villosus DSM 22612 (2)*	55.07	1.75	100.97	100.97	100.97
*M. villosus DSM 22612 (2)*	67.20	1.60	100.60	100.60	100.60
*M. villosus DSM 22612 (2)*	72.93	1.87	99.87	99.87	99.87
*M. villosus DSM 22612 (2)*	83.63	1.45	100.16	100.16	100.16
*M. okinawensis DSM 14208*	19.68	1.36	95.88	95.89	95.89
*M. okinawensis DSM 14208*	45.93	1.48	97.47	97.46	97.46
*M. okinawensis DSM 14208*	63.45	1.53	97.54	97.54	97.54
*M. okinawensis DSM 14208*	93.35	1.48	100.22	100.22	100.22
*M. okinawensis DSM 14208*	116.15	1.58	98.33	98.32	98.32
*M. okinawensis DSM 14208*	137.67	1.62	98.06	98.06	98.06
*M. okinawensis DSM 14208*	158.65	1.57	98.59	98.58	98.58
*M. okinawensis DSM 14208*	177.53	1.55	100.00	100.01	100.01
*M. soligelidi DSM 26065*	120.00	2.67	81.70	81.64	81.64
*M. soligelidi DSM 26065*	236.83	2.83	81.35	81.35	81.35
*M. soligelidi DSM 26065*	351.92	2.21	86.45	86.48	86.48
*M. soligelidi DSM 26065*	478.25	1.33	95.18	95.23	95.23
*M. soligelidi DSM 26065*	589.80	2.01	100.39	100.34	100.34
*M. soligelidi DSM 26065*	715.77	1.69	94.25	94.20	94.20
*M. soligelidi DSM 26065*	831.03	0.94	100.00	99.94	99.94

**Table 3:** **Mass balances of the two experiments with OD measurements**.

**Strain**	**t [h]**	**C-balance (%)**	**H-balance (%)**	**DoR-balance (%)**	**C-balance incl. x (%)**	**H-balance incl. x (%)**	**DoR-balance incl. x (%)**
*M. marburgensis DSM 2133 (65°C)*	13.68	100.00	201.92	99.99	98.76	201.62	99.33
*M. marburgensis DSM 2133 (65°C)*	35.23	100.00	96.32	100.01	105.51	97.62	102.93
*M. marburgensis DSM 2133 (65°C)*	56.45	100.00	92.44	100.00	105.80	93.80	103.08
*M. marburgensis DSM 2133 (65°C)*	77.35	100.00	93.12	100.00	105.34	94.37	102.83
*M. marburgensis DSM 2133 (65°C)*	137.90	100.00	105.73	100.00	113.43	108.88	107.12
*M. marburgensis DSM 2133 (65°C)*	160.57	100.00	103.90	100.00	101.21	104.19	100.64
*M. marburgensis DSM 2133 (65°C)*	180.47	100.00	102.40	100.00	103.46	103.22	101.83
*M. marburgensis DSM 2133 (65°C)*	198.55	100.00	108.25	100.00	101.87	108.69	100.99
*M. villosus DSM 22612 (2)*	11.83	100.00	97.00	100.00	107.93	98.87	89.69
*M. villosus DSM 22612 (2)*	18.82	100.00	97.34	100.00	106.87	98.96	95.97
*M. villosus DSM 22612 (2)*	30.82	100.00	98.03	100.00	102.47	98.60	97.37
*M. villosus DSM 22612 (2)*	37.62	100.00	103.46	100.00	100.46	103.57	95.72
*M. villosus DSM 22612 (2)*	48.20	100.00	102.52	100.00	102.98	103.22	98.74
*M. villosus DSM 22612 (2)*	55.07	100.00	99.47	100.00	101.79	99.89	99.91
*M. villosus DSM 22612 (2)*	67.20	100.00	99.40	100.00	100.84	99.60	101.70
*M. villosus DSM 22612 (2)*	72.93	100.00	104.63	100.00	100.89	104.84	103.88
*M. villosus DSM 22612 (2)*	83.63	100.00	96.81	100.00	102.01	97.28	101.70

In Tables 4, 5 the respective volumetric uptake and evolution rates and mass balances for each experimental run of the OD sampling experiments with *M. marburgensis* and *M. villosus* are shown. The first row (*pressure* + *weight*) represents the values of the method presented in this article. The values of the second row (*pressure* + *weight* + *OD*) include the biomass contribution calculated by the use of the OD data, and for the third row (*weight* + *OD* + *GC*) the GC measurements were taken into account.

**Table 4:** **Comparison of the volumetric uptake and production rates and mass balances for the three methods for ***M. marburgensis*** DSM 2133 (65°C, OD)**.

	**WER**	**MER**	**CUR**	**HUR**	**C-balance (%)**	**H-balance (%)**	**DoR-balance (%)**
Pressure + weight	3.62	1.15	−1.15	−6.21	100.00	108.25	100.00
Pressure + weight + OD	3.62	1.15	−1.15	−6.21	101.87	108.69	100.99
Weight + OD + GC	3.62	1.41	−1.36	−5.67	105.60	113.85	100.26

**Table 5:** **Comparison of the volumetric uptake and production rates and mass balances for the three methods for ***M. villosus*** DSM 22612 (OD)**.

	**WER**	**MER**	**CUR**	**HUR**	**C-balance (%)**	**H-balance (%)**	**DoR-balance (%)**
Pressure + weight	5.68	3.03	−3.03	−12.13	100.00	96.81	100.00
Pressure + weight + OD	5.68	3.03	−3.03	−12.13	102.01	97.28	101.70
Weight + OD + GC	5.68	2.86	−2.59	−11.73	113.10	97.72	98.74

## 5. Discussion

### 5.1. Uncertainty analysis

Several points have been included into the uncertainty analysis. First, when flushing the serum bottles, by inserting a needle, little droplets of the medium might stick to the bottom of the rubber stopper. These droplets could exit the serum bottles which would cause a decrease in mass. Therefore, we used a 3-way-stopcock to avoid this effect. Here, we purge and flush the bottles with the same needle which reduces losing medium/suspension during subsequent flush and purge cycles. If, however, a droplet still exits the flask for example when pulling out the needle this mass data point has to be neglected in the analysis and removed from data treatment procedure (see source code in the Supplementary Material). Another issue concerns the pressure difference during the gassing events and specifically the fact that there might be a small difference in mass when the pressure after gassing is slightly different than the day before (see Supplementary Material, source code).

As already mentioned before, the values in Equations (7) and (12) were experimentally determined. These values were determined for *M. marburgensis* and therefore could vary for other strains. Further experiments have to be done to determine the strain specific values, but the difference in the results should not change significantly due to the fact that the influence of the biomass to the mass balances is rather small.

As can be seen in Tables 2 and 3 (as well as in Tables 1 and 2 of the Supplementary Material) the first two to three data points of each experiment do not match with the subsequent values. This could have several reasons, e.g. that there is some kind of growth lag phase in the beginning or that the yield is somehow shifted. In any case, we would advise to treat the first two data points of each experiment with caution.

### 5.2. Comparison of gained mass values

**Figure 6:**
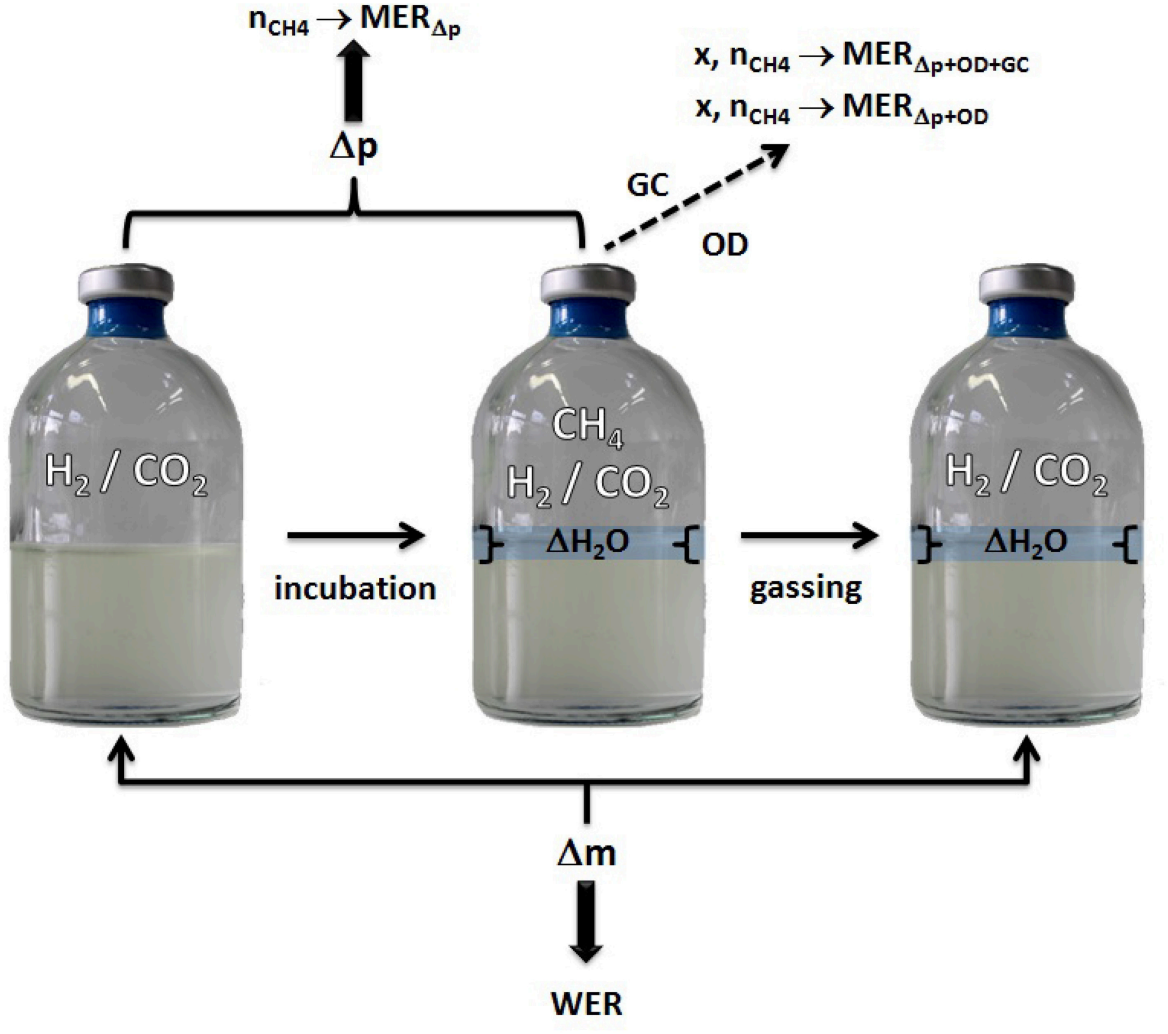
**Model for WER and MER determination using serum bottle headspace pressure and Δ water mass quantification**.

To calculate the mass of the produced H_2_O, several different data values can be used (see also Figure 6): change in pressure, mass increase, and (if available) GC data. As mentioned in Section 3, the change in pressure over the incubation period can be used to calculate the particle number of produced CH_4_. If GC data are available, the percent by volume of CH_4_ is equal to the conversion factor of CO_2_ to CH_4_. Therefore, the number of produced CH_4_ can be calculated. As can be seen in Equation 1, the number of produced CH_4_ is half of the number of the produced H_2_O in mol. In Table 1 of the Supplementary Material the comparison of the results is presented and as can be seen, the discrepancy is very small.

### 5.3. Comparison of mass balances

As can be seen in Tables 4 and 5, the difference in the mass balances between the methods are very small. This leads to the conclusion that additional OD measurements are not necessary to determine the balances due to the fact that the contribution of biomass to these parameters is negligible. Therefore, even though we have not determined the exact elementary composition and degree of reduction of the strains other than *M. marburgensis*, these values do not have a significant influence on the balances. The discrepancy in the C-balances between the GC data and the others might lie in the fact that the used media contained hydrogen carbonates. This could lead to a shift in the CO_2_ consumption and therefore in the composition of the gas phase because full conversion of the substrates could not be performed.

### 5.4. General discussion

The utilization of an anaerobic chamber for the preparation of the media and for inoculation would be helpful but it is not a prerequisite. Alternatively, the aforementioned anaerobic working steps could be performed by either using gas-filled/flushed syringes or by application of an argon bath. The application of resazurin to the medium would only indicate whether the system is above or below a certain oxidation-reduction potential but does not prevent the media from becoming aerobic.

The dependence of the serum bottle temperature on the gas phase headspace pressure and the cooling time was determined experimentally (data not shown). A cooling time of more than 45 min at room temperature is needed to receive proper values for a liquid volume of about 50 mL. However, a cooling time of 1 h would be recommended.

Gas chromatographic measurements in addition to OD measurements were performed at the end of some of the experiments. Therefore, only end point values are available for the mass balance calculations including the GC measurements. The lack of GC data values throughout the experiment demonstrates another advantage of the presented method, namely the possibility of regular screening of the CH_4_ production. For the experiment with *M. soligelidi* the incubation time of 5 days might have been too short, as this time is equal to the doubling time of the strain (Wagner et al., 2013). Hence, it would be necessary to investigate the influence of the gassing pressure or gassing frequency. This method can also be used in the case of turbid media (e.g., *M. soligelidi*), for which spectrophotometric measurement of the optical density is not feasible. Furthermore, the method can also be applied if the determination of optical density is not feasible due to low cell density or in case aggregate forming organisms should be analyzed.

Eventually, this method could also be applied to quantify the production of metabolic end products from non-methanogenic pure cultures—e.g., acetate production by acetogens from CO_2_ and H_2_ in order to be able to quantify gas to liquid conversion rates.

## 6. Conclusions

In order to be able to effectively screen pure cultures of hydrogenotrophic methanogens regarding their CH_4_ production potential we developed a novel method for indirect quantification of CH_4_ production via H_2_O production. This novel method was established in serum bottles for cultivation of hydrogenotrophic methanogens in closed batch cultivation mode. Water production was quantified by determining the difference in mass increase in a quasi-isobaric setting. We demonstrated that this method for the indirect CH_4_ quantification is an extremely accurate and precise technique suited to rapidly screen pure culture of methanogens regarding their CH_4_ production potential, especially if applied in high throughput screening experiments. We conclude that this novel method is a cost effective alternative to determine CH_4_ production potential of methanogens over CH_4_ quantification by using gas chromatography. We show that this method can be universally applied for quantification of CH_4_ production from psychrophilic, thermophilic and hyperthermophilic hydrogenotrophic methanogens.

## Author contributions

SR conceived the experiments. RT and SR designed the experiments. RT performed the experiments. RT and SR analyzed the data. RT wrote the source code. SR supervised research. RT and SR wrote the manuscript. RT and SR approved the final version of the manuscript.

## Funding

RT acknowledges funding by the University of Vienna for her PhD in the frame of the Research Platform: ExoLife (FPF-234). This article was supported by the Open Access Publishing Fund of the University of Vienna.

### Conflict of interest statement

The authors declare that the research was conducted in the absence of any commercial or financial relationships that could be construed as a potential conflict of interest.

## Abbreviations

*CH*_4_: methane
*H*_2_*O*: water
*H*_2_: molecular hydrogen
*CO*_2_: carbon dioxide
*x* [g L^−1^]: biomass concentration
*MER* [mmol h^−1^ L^−1^]: methane evolution rate
*WER* [mmol h^−1^ L^−1^]: water evolution rate
*HUR* [mmol h^−1^ L^−1^]: hydrogen uptake rate
*CUR* [mmol h^−1^ L^−1^]: carbon dioxide uptake rate
*DoR*: Degree of Reduction
*C*−*mol*: moles of carbon
*Y*_(*x/CH*4)_ [C-mol mol^−1^]: growth to product yield
*r*_*x*_ [C-mmol h^−1^ L^−1^]: biomass production rate.
