# Supplementary material for: Method for Indirect Quantification of CH4 Production via H2O Production Using Hydrogenotrophic Methanogens
Source: Front Microbiol. 2016 Apr 29;7:532. doi: 10.3389/fmicb.2016.00532 (PMC4850170; doi:10.3389/fmicb.2016.00532)
Supplement: Supplementary file 1 [file DataSheet1.pdf]

Supplementary Material: Method for indirect quantification of CH_4 production via H_2O production using hydrogenotrophic methanogens

Ruth-Sophie Taubner and Simon K.-M. R. Rittmann*

*Correspondence:
Simon K.-M. R. Rittmann
simon.rittmann@univie.ac.at

1 SOURCE CODE

In the following section, the source code of the data analysing program written in MATLAB is presented. This file is appropriate for analysing a triplicate experiment plus a zero control. All explanations are proceeded with "%".

```
function [MER,WER,DeltaMass_p]=H2O_Exp(T1)

w=dlmread('H2ODATA.txt');   %Reads data from data sheet

V_med=50;  %volume of the liquid media in ml

[m,n]=size(w);  %n-1: number of bottles

x=(m-3)/4;  % number of runs

V=w(1,2:n);   %Volume in cm^3

for i=1:x;
     mass1(i,1:n-1)= w(4+4*(i-1),2:n);  %mass before gassing
     mass2(i,1:n-1)= w(7+4*(i-1),2:n);  %mass after gassing
     t1(i,1)=w(4+4*(i-1),1);     % time at first mass determination in h
     t2(i,1)=w(7+4*(i-1),1);     % time at second mass determination in h
     p_1(i,1:n-1)= w(5+4*(i-1),2:n)+1;            % pressure before gassing
     p_2(i,1)= w(2+4*(i-1),2)+1;            % pressure after gassing
end
p_2=[p_2;w(2+4*x,2)+1];   %pressure after gassing

for i=1:x;
    for j=1:n-1;
if p_1(i,j) ≥ p_2(i,1)
   p_2(i,1)=max(p_1(i,:));
else
end
    end
end
```

```

DeltaMass=mass2-mass1;
for i=2:x;
   MassSum(1,:)=DeltaMass(1,:);
   MassSum(i,:)=MassSum(i-1,:)+DeltaMass(i,:);
end


for i=1:x;
    for j=1:n-1;
p_CH4(i,j)=(p_2(i)-p_1(i,j))/4*10^5            % CH4-partial pressure in Pascal
p_CO2(i,j)=p_2(i)/5*10^5-p_CH4(i,j);           % CO2-partial pressure in Pascal
Konversion(i,j)=p_CH4(i,j)/p_CO2(1)/10^5*100;
     end
end


%ideal Gas equation:
V=V*10^-6;    %in m^3

T=T1+273.15; %K
R=8.3144621; %J/(mol K)

for i=1:x
    for j=1:n-1
n_ref_CO2(i,j)=((V(i,j)*p_2(i)/5*10^5)/(R*T));
n_CH4(i,j)=((V(i,j)*p_CH4(i,j))/(R*T)); %mol   theoretical particle number in mol (based on ...
    pressure drop)
n_H2(i,j)=((V(i,j).*4*p_CO2(i,j))/(R*T)); %mol   theoretical particle number in mol (based on ...
    pressure drop)
n_CO2(i,j)=((V(i,j).*p_CO2(i,j))/(R*T)); %mol   theoretical particle number in mol (based on ...
    pressure drop)
m_H2O(i,j)=2*n_CH4(i,j)*18.01528*1000; %mg    theoretical water production in mg (based on ...
    pressure drop)
V(i+1,j)=V(i,j)-m_H2O(i,j)*10^(-9); %m^3  theoretical decrease in volume in m^3 (based on ...
    pressure drop)
    end
end

%%%%%%%%%%%%%%%%%%%%%%%%%%%%%%%%%%%%%%%%%%%%%%%%%%%%%%%%%%%%%%%%%%%
%MER:
Delta_t=t1-[0;t1(1:end-1)];
Delta_n_CH4=n_CH4;
Delta_n_H2=n_H2-4*n_ref_CO2(i,j);
Delta_n_CO2=n_CO2-n_ref_CO2(i,j);

for i=1:x
    for j=1:n-1
 MER(i,j)=Delta_n_CH4(i,j)*1000/(Delta_t(i))/V(i,j)/10^3; %mmol/h/L
 HUR(i,j)=Delta_n_H2(i,j)*1000/(Delta_t(i))/V(i,j)/10^3; %mmol/h/L
 CUR(i,j)=Delta_n_CO2(i,j)*1000/(Delta_t(i))/V(i,j)/10^3; %mmol/h/L
    end
end
%%%%%%%%%%%%%%%%%%%%%%%%%%%%%%%%%%%%%%%%%%%%%%%%%%%%%%%%%%%%%%%%%%%
```

```

%WER:

for i=1:x
    for j=1:n-1
p_neu(i,j)=1/(8.314472/(0.8*2.01588+0.2*16.0425)*10^3*T)*(p_2(i+1)*10^5.*V(i+1,j)-p_2(i)*10^5.*V(i,j))*10^3; ..
      % in g   m=pV/(R_s*T), where R_s==R/(0.8H2+0.2CO2)
DeltaMass_p(i,j)=DeltaMass(i,j)-p_neu(i,j)-n_CH4(i,j)*16.043;      %in g    including mass ...
    difference due to variations in pressure from one run to the other
    end
end

faktor_Med=1000/V_med;    %factor to scale media volume to 1 liter

for i=1:x
    for j=1:n-1
WER(i,j)=DeltaMass_p(i,j)*faktor_Med*1000/(18.01528*Delta_t(i));   %WER in mmol/h/l
    end
end

for i=1:x
MER_mean(i)=mean(MER(i,2:4));
WER_mean(i)=mean(WER(i,2:4));
HUR_mean(i)=mean(HUR(i,2:4));
CUR_mean(i)=mean(CUR(i,2:4));
% HUR_mean(i)=4*MER_mean(i);
% CUR_mean(i)=MER_mean(i);
Y_CH4_CO2(i)=MER_mean(i)/CUR_mean(i);
Y_CH4_H2(i)=MER_mean(i)/HUR_mean(i);
Y_H2O_H2(i)=WER_mean(i)/HUR_mean(i);
end

MER_mean=MER_mean';
WER_mean=WER_mean';
HUR_mean=HUR_mean';
CUR_mean=CUR_mean';

%%%%%%%%%%%%%%%%%%%%%%%%%%%%%%%%%%%%%%%%%%%%%%%%%%%%%%%%%%%%%%%%%%%%%%%%%%%%
fh1=figure(1);
set(fh1, 'color', 'white');
set(gca,'fontweight','bold','fontsize',16);

plot(t1(1:end-1),DeltaMass_p(1:end-1,:),'o-');

title(['\Delta mass']);
xlabel('time [h]');
ylabel(['\Delta mass [g]']);

hleg1=legend(['NK'],['I'],['II'],['III']);
set(hleg1,'Location','eastoutside');

%%%%%%%%%%%%%%%%%%%%%%%%%%%%%%%%%%%%%%%%%%%%%%%%%%%%%%%%%%%%%%%%%%%%%%%%%%%%

end
```

2 SUPPLEMENTARY DATA

Table 1. Comparison of water quantification via pressure and mass gain for the experiments without OD measurements.

Strain	t [h]	$\Delta m_{\Delta p}$	Δm_{weight}	$\Delta m_{\Delta p}$ / Δm_{weight}
M. marburgensis DSM 2133 (55°C)	*13.68*	5.27	25.78	20.45%
M. marburgensis DSM 2133 (55°C)	*35.23*	2.34	11.77	19.87%
M. marburgensis DSM 2133 (55°C)	*56.45*	5.95	9.28	64.12%
M. marburgensis DSM 2133 (55°C)	*77.35*	15.61	10.53	148.30%
M. marburgensis DSM 2133 (55°C)	*137.90*	19.19	28.22	67.99%
M. marburgensis DSM 2133 (55°C)	*160.57*	44.21	40.24	109.84%
M. marburgensis DSM 2133 (55°C)	*180.47*	24.72	32.12	76.95%
M. marburgensis DSM 2133 (55°C)	*198.55*	28.19	32.09	87.84%
M. marburgensis DSM 2133 (65°C)	*13.68*	5.24	25.02	20.94%
M. marburgensis DSM 2133 (65°C)	*35.23*	23.56	26.47	89.02%
M. marburgensis DSM 2133 (65°C)	*56.45*	44.60	38.33	116.35%
M. marburgensis DSM 2133 (65°C)	*77.35*	43.25	38.88	111.24%
M. marburgensis DSM 2133 (65°C)	*137.90*	41.41	44.69	92.66%
M. marburgensis DSM 2133 (65°C)	*160.57*	44.79	47.17	94.94%
M. marburgensis DSM 2133 (65°C)	*180.47*	43.60	45.30	96.24%
M. marburgensis DSM 2133 (65°C)	*198.55*	45.84	51.79	88.52%
M. villosus DSM 22612 (1)	*19.68*	43.08	43.19	99.76%
M. villosus DSM 22612 (1)	*45.93*	43.83	42.05	104.24%
M. villosus DSM 22612 (1)	*63.45*	43.30	45.26	95.67%
M. villosus DSM 22612 (1)	*93.35*	44.43	46.62	95.30%
M. villosus DSM 22612 (1)	*116.15*	45.71	46.67	97.94%
M. villosus DSM 22612 (1)	*137.67*	44.65	47.09	94.80%
M. villosus DSM 22612 (1)	*158.65*	44.41	46.55	95.41%
M. villosus DSM 22612 (1)	*177.53*	44.67	48.76	91.62%
M. villosus DSM 22612 (2)	*11.83*	45.35	46.04	98.50%
M. villosus DSM 22612 (2)	*18.82*	48.12	49.16	97.89%
M. villosus DSM 22612 (2)	*30.82*	49.31	48.37	101.95%
M. villosus DSM 22612 (2)	*37.62*	48.59	50.52	96.17%
M. villosus DSM 22612 (2)	*48.20*	48.84	52.48	93.07%
M. villosus DSM 22612 (2)	*55.07*	45.44	50.57	89.86%
M. villosus DSM 22612 (2)	*67.20*	48.00	50.76	94.56%
M. villosus DSM 22612 (2)	*72.93*	39.75	46.07	86.28%
M. villosus DSM 22612 (2)	*83.63*	51.24	51.28	99.92%
M. okinawensis DSM 14208	*19.68*	42.54	41.99	101.30%
M. okinawensis DSM 14208	*45.93*	42.69	44.19	96.59%
M. okinawensis DSM 14208	*63.45*	41.86	44.23	94.63%
M. okinawensis DSM 14208	*93.35*	44.53	46.16	96.47%
M. okinawensis DSM 14208	*116.15*	41.24	44.35	93.00%
M. okinawensis DSM 14208	*137.67*	38.82	42.36	91.63%
M. okinawensis DSM 14208	*158.65*	39.13	42.05	93.04%
M. okinawensis DSM 14208	*177.53*	37.90	40.43	93.74%
M. soligelidi DSM 26065	*120.00*	15.12	22.84	66.21%
M. soligelidi DSM 26065	*236.83*	14.61	22.99	63.54%
M. soligelidi DSM 26065	*351.92*	15.24	20.22	75.38%
M. soligelidi DSM 26065	*478.25*	14.06	13.72	102.46%
M. soligelidi DSM 26065	*589.80*	13.10	16.37	80.06%
M. soligelidi DSM 26065	*715.77*	14.41	16.14	89.26%
M. soligelidi DSM 26065	*831.03*	11.44	9.42	121.49%

Table 2. Comparison of water quantification via pressure, mass gain and GC for the experiments with OD measurements.

Strain	t [h]	$\Delta m_{\Delta p}$	Δm_{weight}	Δm_{GC}	$\Delta m_{\Delta p}$ / Δm_{weight}	Δm_{GC} / Δm_{weight}
M. marburgensis DSM 2133 (65°C)	*13.68*	6.35	19.29		303.81%	
M. marburgensis DSM 2133 (65°C)	*35.23*	15.80	14.64		92.63%	
M. marburgensis DSM 2133 (65°C)	*56.45*	46.06	39.09		84.86%	
M. marburgensis DSM 2133 (65°C)	*77.35*	45.17	38.95		86.23%	
M. marburgensis DSM 2133 (65°C)	*137.90*	37.50	41.79		111.42%	
M. marburgensis DSM 2133 (65°C)	*160.57*	44.40	47.87		107.81%	
M. marburgensis DSM 2133 (65°C)	*180.47*	43.36	45.44		104.79%	
M. marburgensis DSM 2133 (65°C)	*198.55*	45.86	53.42	41.59	116.48%	77.85%
M. villosus DSM 22612 (2)	*11.83*	45.90	43.15		94.01%	
M. villosus DSM 22612 (2)	*18.82*	48.49	45.91		94.68%	
M. villosus DSM 22612 (2)	*30.82*	50.82	48.81		96.05%	
M. villosus DSM 22612 (2)	*37.62*	44.77	47.87		106.92%	
M. villosus DSM 22612 (2)	*48.20*	47.26	49.64		105.04%	
M. villosus DSM 22612 (2)	*55.07*	48.02	47.51		98.95%	
M. villosus DSM 22612 (2)	*67.20*	53.17	52.52		98.79%	
M. villosus DSM 22612 (2)	*72.93*	39.43	43.07		109.25%	
M. villosus DSM 22612 (2)	*83.63*	54.28	50.82	51.29	93.62%	100.94%

3 SUPPLEMENTARY TABLES AND FIGURES

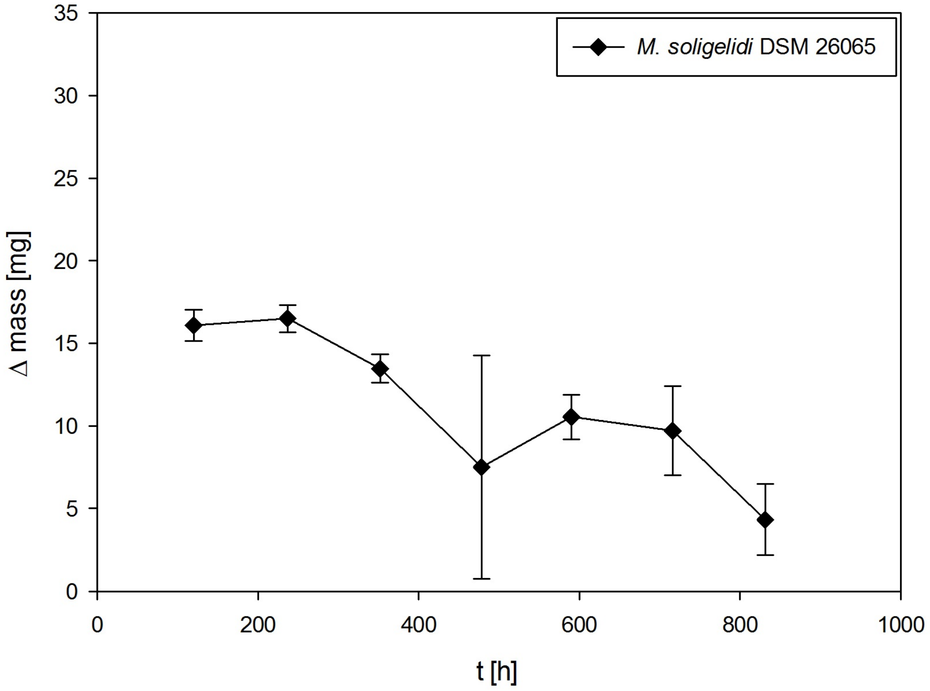

Figure 1. Mass gain of *M. soligelidi*. Negative control is not shown.

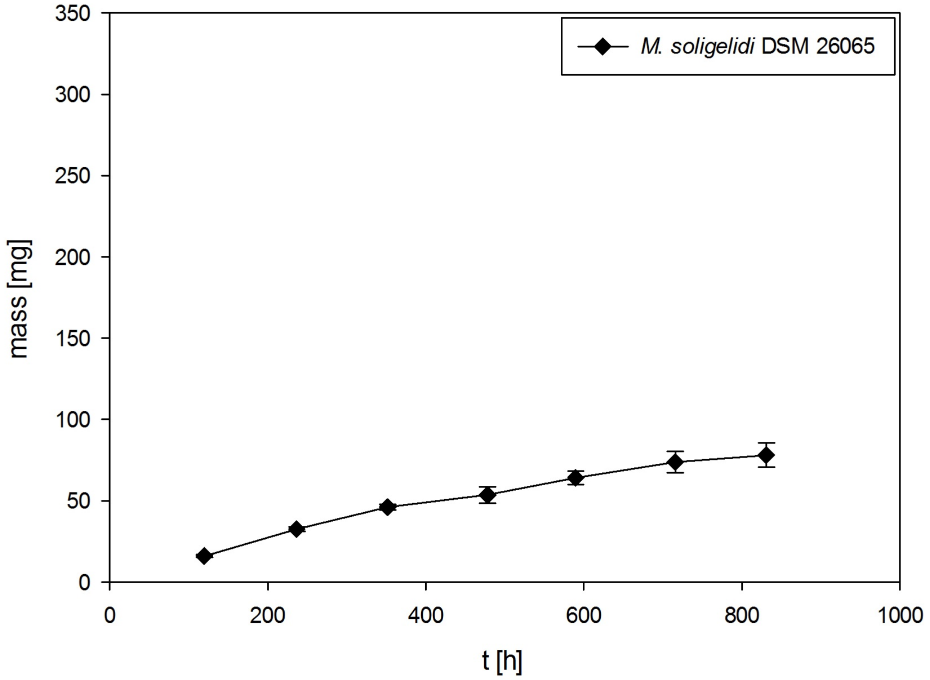

Figure 2. Total cumulative mass gain of *M. soligelidi*. Negative control is not shown.

Date	Measurement	time start	time end	Δ t	T [°C] before	T [°C] after	F45_ZC	F45_I	F45_II	F45_III	F55_ZC	F55_I	F55_II	F55_III	F65_ZC	F65_I	F65_II	F65_III
16.02.2015	mass [g] (0.8bar)																	
	mass [g]																	
	Pressure [bar] before																	
	Pressure [bar] after																	
	Pressure [bar] Reference Flask																	
	mass [g]																	
	difference (1 day)																	
	difference (2 days)																	
17.02.2015	mass [g]																	
	Pressure [bar] before																	
	Pressure [bar] after																	
	Pressure [bar] Reference Flask																	
	mass [g]																	
	difference (1 day)																	
	difference (2 days)																	
18.02.2015	mass [g]																	
	Pressure [bar] before																	
	Pressure [bar] after																	
	Pressure [bar] Reference Flask																	
	mass [g]																	
	difference (1 day)																	
	difference (2 days)																	
19.02.2015	mass [g]																	
	Pressure [bar] before																	
	Pressure [bar] after																	
	Pressure [bar] Reference Flask																	
	mass [g]																	
	difference (1 day)																	
	difference (2 days)																	
20.02.2015	mass [g]																	
	Pressure [bar] before																	
	Pressure [bar] after																	
	Pressure [bar] Reference Flask																	
	mass [g]																	
	difference (1 day)																	
	difference (2 days)																	
23.05.2015	mass [g]																	
	Pressure [bar] before																	
	Pressure [bar] after																	
	Pressure [bar] Reference Flask																	
	mass [g]																	
	difference (1 day)																	

Supplementary Table 3. Blank data sheet for six experimental runs for a triplicate experiment for three different temperatures.

Supplementary Table 4. Example for input file for a triplicate experiment of the MATLAB file above – the first line and the first column (written text) should not be included into the file H2ODATA.txt

	time[h]	Zero Control	Bottle 1	Bottle 2	Bottle 3
mass [g]	0.00	68.0378	62.7352	62.7091	62.7126
pressure [barg]	0.00	1.5360			
mass [g]	0.00	147.0002	151.4489	152.3016	151.8365
mass [g]	18.88	146.9990	151.4493	152.3010	151.8367
pressure [barg]	18.88	1.5170	-0.3340	-0.3390	-0.3420
pressure [barg]	18.88	1.5690			
mass [g]	18.88	147.0082	151.4924	152.3427	151.8793
mass [g]	45.15	147.0089	151.4955	152.3447	151.8813
pressure [barg]	45.15	1.5770	-0.3070	-0.3100	-0.2990
pressure [barg]	45.15	1.5820			
mass [g]	45.15	147.0106	151.5396	152.3889	151.9257
mass [g]	62.53	147.0091	151.5390	152.3882	151.9240
pressure [barg]	62.53	1.5680	-0.2730	-0.2560	-0.2660
pressure [barg]	62.53	1.6350			
mass [g]	62.53	147.0132	151.5847	152.4325	151.9690
mass [g]	92.87	147.0125	151.5861	152.4323	151.9696
pressure [barg]	92.87	1.6470	-0.3240	-0.3200	-0.3140
pressure [barg]	92.87	1.5900			
mass [g]	92.87	147.0116	151.6329	152.4767	152.0153
mass [g]	115.62	147.0112	151.6334	152.4774	152.0165
pressure [barg]	115.62	1.6050	-0.2100	-0.2700	-0.1720
pressure [barg]	115.62	1.6030			
mass [g]	115.62	147.0113	151.6786	152.5225	152.0591
mass [g]	137.25	147.0103	151.6786	152.5221	152.0587
pressure [barg]	137.25	1.5900	-0.1200	-0.1380	-0.0820
pressure [barg]	137.25	1.6070			
mass [g]	137.25	147.0100	151.7216	152.5648	152.1005
mass [g]	157.53	147.0118	151.7239	152.5666	152.1027
pressure [barg]	157.53	1.6160	-0.1730	-0.0830	-0.0890
pressure [barg]	157.53	1.6490			
mass [g]	157.53	147.0122	151.7681	152.6076	152.1448
mass [g]	177.00	147.0109	151.7679	152.6071	152.1436
pressure [barg]	177.00	1.6490	-0.0600	-0.0210	-0.0050
pressure [barg]	177.00	1.5960			
mass [g]	177.00	147.0082	151.8090	152.6466	152.1823
